# Supplementary material for: GPS Coordinates for Modelling Correlated Herd Effects in Genomic Prediction Models Applied to Hanwoo Beef Cattle
Source: Animals (Basel). 2021 Jul 9;11(7):2050. doi: 10.3390/ani11072050 (PMC8300180; doi:10.3390/ani11072050)
Supplement: Supplementary file 1 [file animals-11-02050-s001.zip › animals-1246779-supplementary.pdf]

**File S1: Supplementary material A**

The Gaussian process regression is a powerful tool used to make spatial inferences of unobserved data, using observed data, by correlating the data through their spatial distances. However, in the context of our work, all herds are accounted for, by a set of observations in the reference population. Therefore, the models we propose do not aim to predict herd effects for herds that are absent in the reference population. We reinforce here that the objective of our study was to evaluate the different results in genomic prediction from modelling random herd effects as either independent or correlated. We will now demonstrate why, in the context of our study, the Gaussian process regression is redundant to the GPS-based distances, therefore yielding results that are mathematically equivalent.

The Gaussian process regression in spatial analysis is defined by the Matérn covariance function applied to the distances ($d$):

$$C_\alpha(d_i) = \sigma^2 \frac{2^{1-\alpha}}{\Gamma(\alpha)}\left(\sqrt{2\alpha}\frac{d_i}{\rho_i}\right)^\alpha K_\alpha\left(\sqrt{2\alpha}\frac{d_i}{\rho_i}\right).$$

$C_\alpha(d)$ is proportional to the product of a power function on the distances, $\left(\sqrt{2\alpha}\frac{d_i}{\rho_i}\right)^\alpha$ and the modified Bessel function of the third kind, $K_\alpha\left(\sqrt{2\alpha}\frac{d_i}{\rho_i}\right): K_\alpha(x) = \frac{\sqrt{\pi}}{\left(\alpha-\frac{1}{2}\right)!}\left(\frac{x}{2}\right)^\alpha \int_0^\infty u^{\alpha-1/2}e^{-x\sqrt{u+1}}du$, one of the many representations of $K_\alpha(x)$ that we can use. We chose this particular representation of $K_\alpha$ because $K_\alpha\left(\sqrt{2\alpha}\frac{d_i}{\rho_i}\right) = \frac{\sqrt{\pi}}{\left(\alpha-\frac{1}{2}\right)!}\left(\frac{\sqrt{2\alpha}}{2}\frac{d_i}{\rho_i}\right)^\alpha \int_0^\infty u^{\alpha-1/2}e^{-\frac{d_i}{\rho_i}\sqrt{2\alpha(u+1)}}du$ directly projects distances defined at the shell of a sphere (such as GPS-based distances at the surface of the globe) into a hyperbolic plane. The hyperbolic plane is a “wavy” plane defined by a mixture of $sinh(x)$ and $cosinh(x)$. This projection converts the problem of maximizing a function defined over a sphere into a problem of maximizing a function that does have more tangible peaks in its 3D visualization.

Now, let us go back to the purpose of all this. The Matérn covariance is used to make inferences of unobserved data, using the observations on regions that surround the area of interest. The projection to the hyperbolic plane allows the search of points that maximizes the likelihood function for this inference of unobserved data. The method follows the synthesized algorithm below:

1. Define the likelihood function using the Matérn covariance as $L(\sigma^2, \alpha, \boldsymbol{\rho}|\boldsymbol{d}, \boldsymbol{y}, \boldsymbol{\eta}_{obs}, \boldsymbol{\eta}_{unobs})$,
2. Initialize $\sigma^2, \alpha, \boldsymbol{\rho}$, $\boldsymbol{\eta}_{obs}$ and $\boldsymbol{\eta}_{unobs}$ as $\hat{\sigma}^{2(0)}, \hat{\alpha}^{(0)}, \hat{\boldsymbol{\rho}}^{(0)}$, $\boldsymbol{\eta}_{obs}^{(0)}$ and $\boldsymbol{\eta}_{unobs}^{(0)}$,
3. Run until convergence:

a. $\hat{\sigma}^{2(t+1)}, \hat{\alpha}^{(t+1)}, \hat{\boldsymbol{\rho}}^{(t+1)} = argmax_{\sigma^2,\alpha,\rho} L(\sigma^2, \alpha, \boldsymbol{\rho} | \boldsymbol{d}, \boldsymbol{y}, \boldsymbol{\eta}_{obs}^{(t)}, \boldsymbol{\eta}_{unobs}^{(t)})$

b. $\boldsymbol{\eta}_{obs}^{(t+1)} \sim f(\boldsymbol{\eta}_{obs} | \boldsymbol{y}, \boldsymbol{\eta}_{unobs}^{(t)}, \hat{\sigma}^{2(t+1)}, \hat{\alpha}^{(t+1)}, \hat{\boldsymbol{\rho}}^{(t+1)})$

c. $\boldsymbol{\eta}_{unobs}^{(t+1)} \sim f(\boldsymbol{\eta}_{unobs} | \boldsymbol{y}, \boldsymbol{\eta}_{obs}^{(t+1)}, \hat{\sigma}^{2(t+1)}, \hat{\alpha}^{(t+1)}, \hat{\boldsymbol{\rho}}^{(t+1)})$

In a situation like ours; in which we are not predicting $\boldsymbol{\eta}_{unobs}$, the algorithm, is shortened to:

1. Define the likelihood function using the Matérn covariance as $L(\sigma^2, \alpha, \boldsymbol{\rho} | \boldsymbol{d}, \boldsymbol{y}, \boldsymbol{\eta}_{obs})$,
2. Initialize $\sigma^2, \alpha, \boldsymbol{\rho}$ and $\boldsymbol{\eta}_{obs}$ as $\hat{\sigma}^{2(0)}, \hat{\alpha}^{(0)}, \hat{\boldsymbol{\rho}}^{(0)}$ and $\boldsymbol{\eta}_{obs}^{(0)}$,
3. Run until convergence:
    a. $\hat{\sigma}^{2(t+1)}, \hat{\alpha}^{(t+1)}, \hat{\boldsymbol{\rho}}^{(t+1)} = argmax_{\sigma^2,\alpha,\rho} L(\sigma^2, \alpha, \boldsymbol{\rho} | \boldsymbol{d}, \boldsymbol{y}, \boldsymbol{\eta}_{obs}^{(t)})$
    b. $\boldsymbol{\eta}_{obs}^{(t+1)} \sim f(\boldsymbol{\eta}_{obs} | \boldsymbol{y}, \hat{\sigma}^{2(t+1)}, \hat{\alpha}^{(t+1)}, \hat{\boldsymbol{\rho}}^{(t+1)})$

The projection of the sphere into the hyperbolic plane define by $K_\alpha$ has its most strong effect in the algorithm in the stage of inferring $\boldsymbol{\eta}_{unobs}^{(t+1)}$. In the absence of this inference at step 3.c, the projection has a weak role in the algorithm and in the course of the iterations its effect will be diluted as the number of iterations increase, resulting in $K_\alpha\left(\sqrt{2\alpha}\frac{d_i}{\rho_i}\right) \underset{t\uparrow}{\longrightarrow} 1$. Moreover, the parameter $\alpha$ is strongly tied to the modified Bessel function $K_\alpha\left(\sqrt{2\alpha}\frac{d_i}{\rho_i}\right)$, and when its projection converges to 1 the projection is actually no longer existent, leaving the distances in the Matérn covariance function within the surface of a sphere. This will put a force into parameter $\alpha$ to "flatten" the power function $\left(\sqrt{2\alpha}\frac{d_i}{\rho_i}\right)^\alpha$ into the spherical surface, thus $\alpha \underset{t\uparrow}{\longrightarrow} 1$. Finally:

$$C_\alpha(d_i) = \sigma^2 \frac{2^{1-\alpha}}{\Gamma(\alpha)}\left(\sqrt{2\alpha}\frac{d_i}{\rho_i}\right)^\alpha K_\alpha\left(\sqrt{2\alpha}\frac{d_i}{\rho_i}\right) \underset{t\uparrow}{\longrightarrow} \hat{\sigma}^2\sqrt{2}\frac{d_i}{\hat{\rho}_i}$$

which is proportional to the GPS-based distances, making the use of the Gaussian process regression equivalent to our proposed herd relationship matrix. Therefore, the results using this method will differ minimally from the results that do not use the Matérn covariance function, and this minimal difference will be only due to the iterative method used to estimate the parameters and effects. Mathematically, in a scenario in which we have observations representing all the herds in the reference data, the results are to be identical.

**File S2: Supplementary material B**

Because we have 124 farms, we have 7,626 unique distances between different farms. To verify empirically the strength of the theoretical results presented in Appendix A, we ran the method in two different scenarios. One scenario where parameter $\alpha$ was assumed the same for all distances, and another scenario where parameter $\alpha$

was assumed different for each distance. To solve this system, we applied a Bayesian approach with flat priors. We ran 75,000 iterations of MCMC, discarding the first 25,000 as burn-in. We present here the summaries for $\alpha$ for both scenarios previously described. We also present the summaries of $K_\alpha\left(\sqrt{2\alpha}\frac{d_i}{\rho_i}\right)$'s, $h^2$, $e^2$, $r_{PGBV}$, and $r_\eta$, for both scenarios considered to estimate parameter $\alpha$.

On the supplementary figures 1 and 2 we could observe that the theoretical expectations over $\alpha$ and $K_\alpha\left(\sqrt{2\alpha}\frac{d_i}{\rho_i}\right)$ were fulfilled. On supplementary figure 3 we could observe that the results for both $h^2$ and $e^2$ did not differ substantially from the results obtained with our REML approach, using the GPS-based relationship matrix to induce correlations between the herds. On supplementary figure 4 we could observe that the results for both $r_{PGBV}$, and $r_\eta$ did not differ substantially from the results obtained with our BLUP approach, using the GPS-based relationship matrix to induce correlations between the herds.

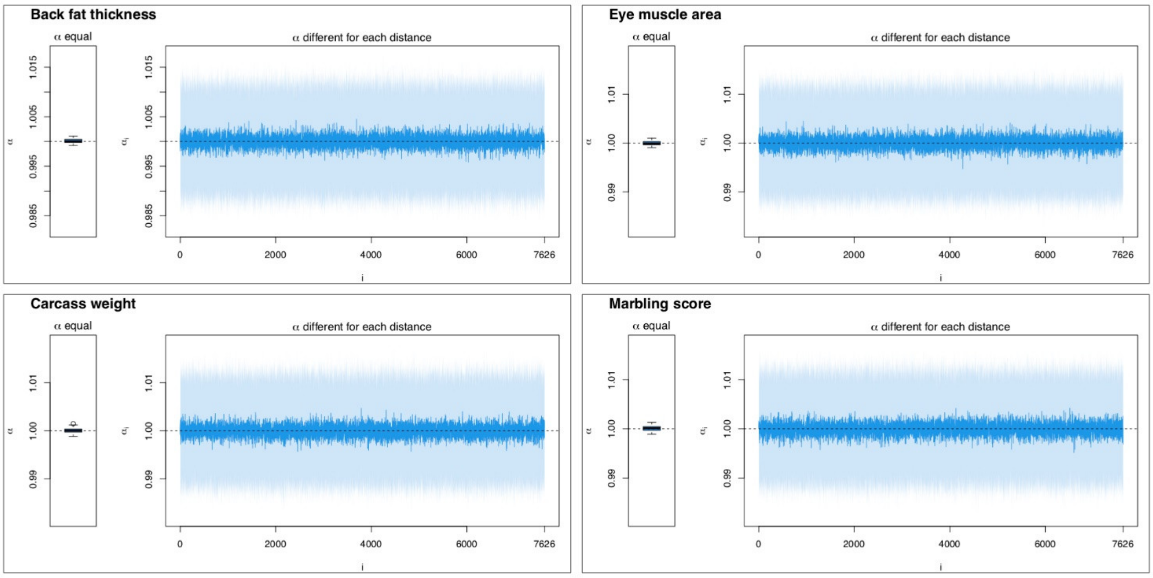


**Figure S1.** Summary of post-Gibbs means of $\alpha$ over all 100 replicates, for all four traits evaluated. For $\alpha$ estimated as the same parameter for all distances, the boxplots are of the post-Gibbs means on the 100 replicates. For $\alpha$ estimated as a different parameter for each distance, the sky-blue points represent $\bar{\alpha}$ and the light blue areas represent $\bar{\alpha} \pm sd_\alpha$ over the results on the 100 replicates.

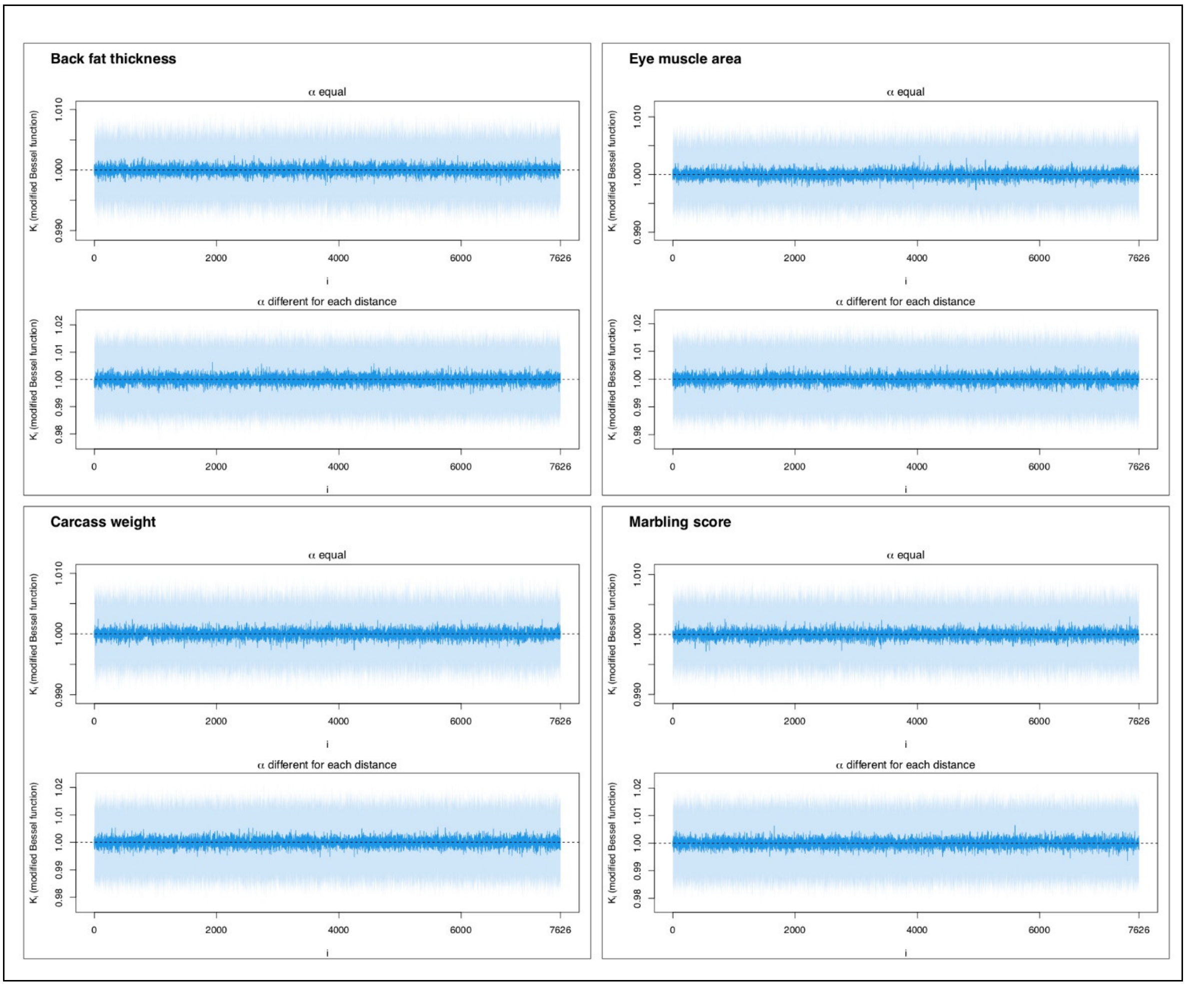


**Figure S2.** Summary of post-Gibbs means of $K_\alpha\left(\sqrt{2\alpha}\frac{d_i}{\rho_i}\right)$ over all 100 replicates, for all four traits evaluated. The sky-blue points represent $\overline{K_\alpha}$ and the light blue areas represent $\overline{K_\alpha} \pm sd_{K_\alpha}$ over the results on the 100 replicates.

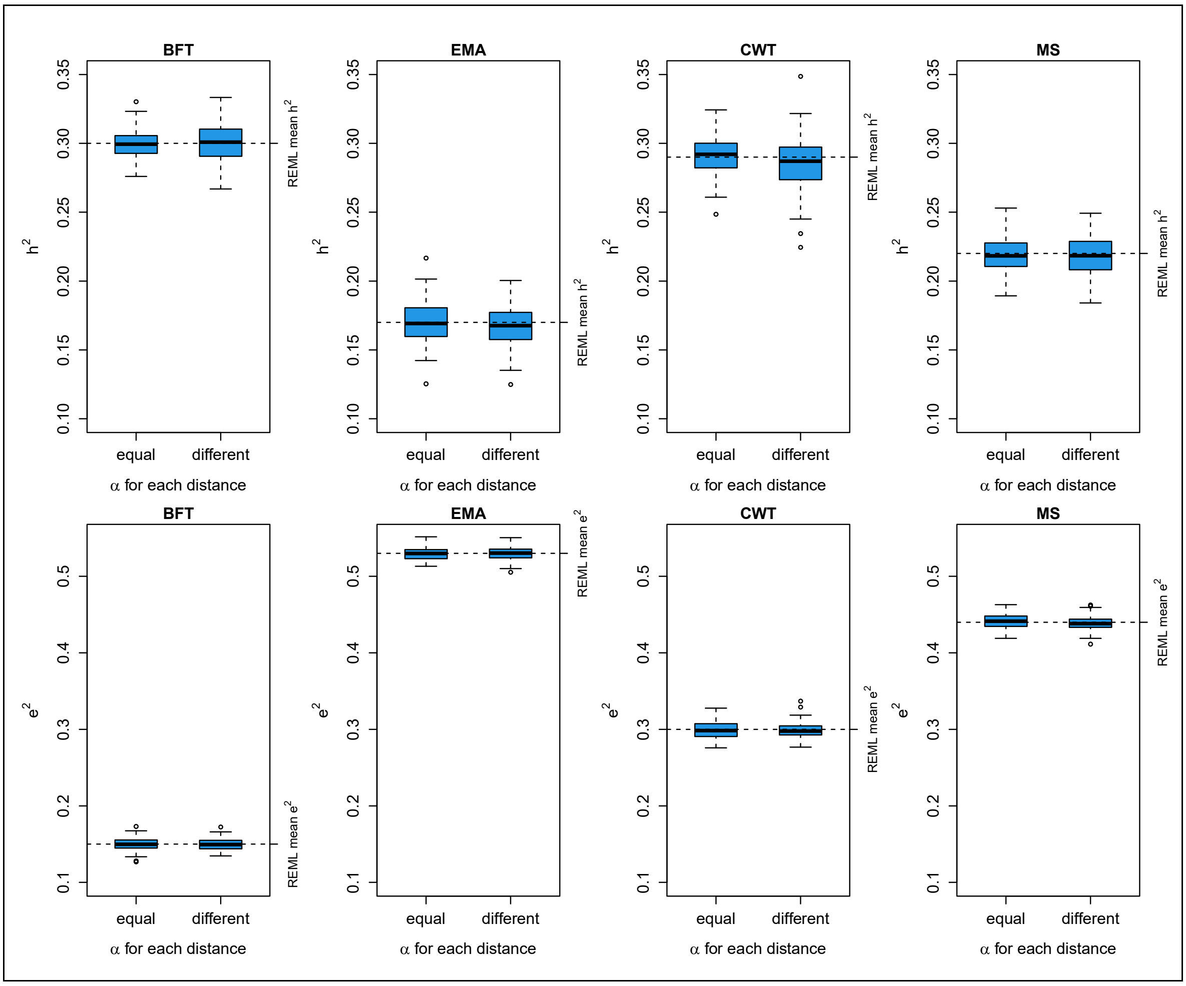


**Figure S3: Summary of post-Gibbs means of $h^2$ and $e^2$ over all 100 replicates, for all four traits evaluated.** Table 100. replicates.

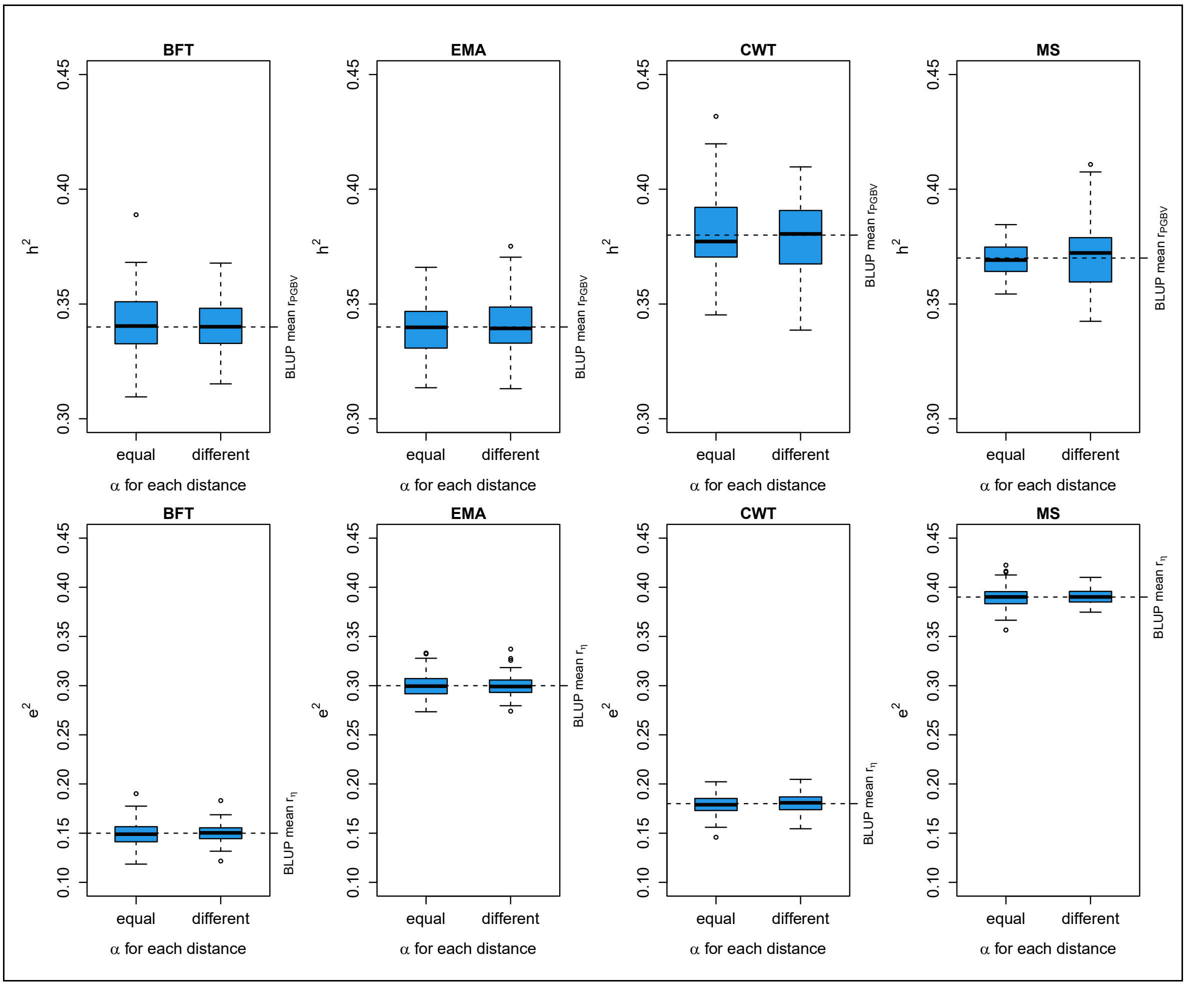


**Figure S4: Summary of post-Gibbs means of $r_{PGBV}$ and $r_\eta$ over all 100 replicates, for all four traits evaluated.** Table 100. replicates.
